# Methodologies for network meta-analysis of randomised controlled trials in pain, anaesthesia, and perioperative medicine: a narrative review

**DOI:** 10.1016/j.bja.2024.12.039

**Published:** 2025-02-19

**Authors:** Brett Doleman, Janus Christian Jakobsen, Ole Mathiesen, Nicola Cooper, Alex Sutton, Jonathan Hardman

**Affiliations:** 1 University of Nottingham, Nottingham, UK; 2 Copenhagen Trial Unit, Centre for Clinical Intervention Research, Capital Region of Denmark & Department of Regional Health Research, The Faculty of Health Sciences, University of Southern Denmark, Odense, Denmark; 3 Centre for Anaesthesiological Research, Department of Anaesthesiology, Zealand University Hospital Køge, Køge, Denmark; 4 Department of Clinical Medicine, Copenhagen University, Copenhagen, Denmark; 5 Complex Reviews Synthesis Unit (CRSU), Biostatistics Research Group, Department of Population Health Sciences, University of Leicester, Leicester, UK

**Keywords:** network meta-analysis, review methodology, statistics in anaesthesia, systematic review, transitivity

## Abstract

Network meta-analysis has emerged as a method for analysing clinical trials, with a large increase in the number of publications over the past decade. Network meta-analysis offers advantages over traditional pairwise meta-analysis, including increased power, the ability to compare treatments not compared in the original trials, and the ability to rank treatments. However, network meta-analyses are inherently more complex than pairwise meta-analyses, requiring additional statistical expertise and assumptions. Many factors can affect the certainty of evidence from pairwise meta-analysis and can often lead to unreliable results. Network meta-analysis is prone to all these issues, although it has the additional assumption of transitivity. Here we review network meta-analyses, problems with their conduct and reporting, and methodological strategies that can be used by those conducting reviews to help improve the reliability of their findings. We provide evidence that violation of the assumption of transitivity is relatively common and inadequately considered in published network meta-analyses. We explain key concepts with clinically relevant examples for those unfamiliar with network meta-analysis to facilitate their appraisal and application of their results to clinical practice.

**Editor's key points**

• Network meta-analyses (NMAs) provide a synthesis of evidence that can be used to inform treatment guidelines, so it is important to ensure that they are conducted and interpreted with the highest standards.
• Network meta-analysis offers many advantages over pairwise meta-analysis, but is inherently more complex, with the important additional assumption of transitivity.
• The authors review evidence from published studies of violations of this assumption, coupled with inadequate conduct of NMAs in the field of anaesthesia and other specialties.
• Approaches to improve the conduct and interpretation of NMAs are critical for the preparation, appraisal, and clinical application of NMAs in pain, anaesthesia, and perioperative medicine.

The number of published network meta-analyses (NMAs) has greatly increased in recent years, increasing by a factor of 20 from 2010 to 2017.^1^ Network meta-analyses can be used to inform treatment guidelines^2^, ^3^, ^4^; it is therefore of utmost importance to ensure that their conduct and reporting are of the highest standards.^5^ Network meta-analysis offers advantages over pairwise meta-analysis (PWMA), including increased power, the ability to compare treatments not compared in the original trials, and the ability to rank treatments.^6^ This review discusses the methodological considerations specific to NMA.

Issues that affect the certainty of evidence in PWMA include risks of bias, random errors, publication bias, and heterogeneity in the included studies.^6^ NMAs can also suffer from all these limitations, which can manifest as poor ability to predict results from large randomised controlled trials (RCTs)^7^ and a scarcity of high-quality evidence.^8^ However, NMA has an additional fundamental assumption known as transitivity, which states that study characteristics that influence the effectiveness of the interventions are balanced between different treatment comparisons. Here we use simple clinical examples from acute pain studies to illustrate principles of NMA with simplified equations aimed at clinicians not familiar with statistics. After discussing what an NMA is and aspects of the methodology that should be considered to reduce erroneous conclusions, we present evidence from meta-epidemiological studies to support arguments for the limitations in the conduct of NMAs, both specific to anaesthesia and for other specialties. We aim to help authors of NMAs improve their methodology and help clinicians appraise such articles to guide their practise.

## What is network meta-analysis?

Network meta-analysis is a meta-analytic technique that estimates treatment effects from direct (_DIR_) and indirect (_IND_) estimates.^9^ Direct effects are obtained when two interventions have been compared within a trial (trial one compares A *vs* B and trial two compares A *vs* C). Indirect effects can then be calculated, even when interventions are not directly compared within a trial (C *vs* B), by computing the difference between each intervention (B *vs* C) and a third intervention, called a common comparator (A).

**Fig 1:**
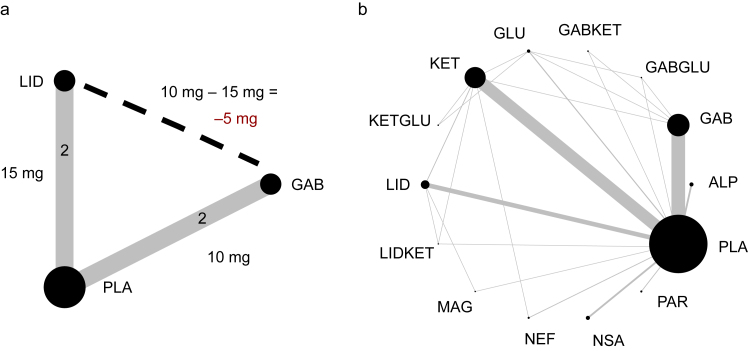
(a) Network plot showing the hypothetical network meta-analysis. Each direct comparison of lidocaine (LID) and gabapentinoids (GAB) with placebo (PLA) is shown. The dashed line shows the indirect comparison between GAB and LID. Using the formulae in the text, an estimated reduction of 5 mg in morphine dose with LID can be achieved based on indirect comparisons derived from direct comparisons with a common comparator. (b) Network plot of interventions for reducing the incidence of chronic postsurgical pain (CPSP).^12^ Compared with (a), there are loops of evidence showing direct and indirect estimates for GAB, ketamine (KET), and PLA. This can be used to assess inconsistency as opposed to the evidence from the same plot for previous interventions as there is no trial comparing these agents directly. ALP, alpha-2 agonists; GAB, gabapentinoids; GABGLU, gabapentinoids and glucocorticoids; GABKET, gabapentinoids and ketamine; GLU, glucocorticoids; KET, ketamine; KETGLU, ketamine and glucocorticoids; LID, lidocaine; LIDKET, lidocaine and ketamine; MAG, magnesium; NEF, nefopam; NSA, NSAIDs and COX-2 inhibitors; PAR, paracetamol; PLA, placebo.

As a specific example, we imagine an NMA of nonopioid analgesics for treatment of acute pain that includes four infinitely large trials that use 24-h morphine consumption as their outcome. Although pain might be a more clinically relevant outcome,^10^ we use morphine consumption as our previous data suggest it has the appropriate statistical characteristics to help illustrate our arguments. Two trials include placebo *vs* gabapentin, whilst the other two trials include placebo *vs* lidocaine (Fig. 1a). Using a PWMA, we can only include mean difference (MD) estimates from trials where interventions are directly compared. However, in an NMA we can compare the effects of both active treatments by looking at the difference between each active drug and placebo. This is given by equation (1). We have excluded sampling error and the possibility of heterogeneity^11^ to help with interpretation of the equations by including infinitely large trials:

(1) MDINDlidocainevsgabapentin=MDDIRplacebovsgabapentin–MDDIRplacebovslidocaine

In this example, we will assume that the effects of gabapentin in two trials are a reduction in morphine consumption of 10 mg compared with placebo; for lidocaine, the reduction compared with placebo is 15 mg. We can estimate the indirect effect of lidocaine *vs* gabapentin as being a reduction of 5 mg in favour of lidocaine using the above formula:

(2) MDINDlidocainevsgabapentin=MDDIRplacebovsgabapentin–MDDIRplacebovslidocaine=10mg–15mg=–5mg

This process can be repeated multiple times in more complex networks to yield multiple indirect estimates via multiple common comparators (Fig. 1b). After this calculation of indirect estimates, both direct (if available) and indirect estimates can be pooled together to give a mixed estimate. By combining two estimates rather than one, statistical power and clinical reliability are potentially increased. Although this can be thought of conceptually and shown mathematically,^13^ this increase in power has also been formally demonstrated empirically.^14^^,^^15^

We briefly discuss the calculation of variance (sampling error) to demonstrate the contribution made by indirect estimates to mixed estimates. For indirect estimates, variance (VAR) is calculated by adding the variances of each of the direct estimates above (2)^15^ This means that the indirect estimate variance will always be higher than the direct estimates they are based on. To illustrate, we assume that the variances from both direct estimates are equal to one; indirect variance is described by:

(3) VARINDlidocainevsgabapentin=VARDIRplacebovsgabapentin+VARDIRplacebovslidocaine=1+1=2

This variance can increase to large values as the number of estimates increases. Alternatively, if we calculate the variance for two studies directly comparing lidocaine and gabapentin using the same variance as the previous example, the variance is lower:

(4) VARDIRlidocainevsgabapentin=(1/1+1/1)–1=0.5

Because of the addition of indirect effects, NMA has advantages over traditional PWMAs, which can only include direct estimates. Firstly, although we have discussed the higher variance of indirect estimates when compared with direct estimates, the addition of indirect estimates contributes additional information so statistical power is improved.^14^^,^^15^ Secondly, NMA allows comparison of agents not compared within the original trials. Although large, well-conducted RCTs are the gold standard for assessing interventions, a large RCT comparing all treatments might be unfeasible and prohibitively expensive, so NMA offers an alternative approach. An example of this would be an NMA examining antidepressant therapy, which included >20 interventions and >100 000 participants; this would be an extremely challenging goal for an RCT.^16^ Thirdly, NMA allows use of statistical techniques that can help identify potential best treatments, such as ondansetron and dexamethasone combinations after intrathecal morphine.^17^ NMA can also include multi-arm trials whilst reducing the risk of double counting in multiple pairwise comparisons.^18^ Finally, NMA can consider the entire evidence base in a systematic fashion and allows formal investigation of inconsistency. For example, if results from multiple PWMAs are compared informally, transitivity can still be an issue (discussed below), but NMA allows this to be investigated and quantified formally, aiding clinical decision-making.

Disadvantages of NMAs include their inherent statistical complexity; it is therefore recommended that NMAs be conducted with a statistician experienced in NMA.^19^ There are also concerns over NMAs being largely informed by indirect evidence. A previous analysis of 213 NMAs found that indirect evidence was responsible for the majority of the information used to inform treatment effects.^20^ There is debate over whether direct evidence should be preferred to indirect estimates. Previous studies have identified problems with low power of indirect estimates.^21^ If indirect effects are calculated from unbiased direct estimates, indirect estimates should also be unbiased (assuming transitivity).^22^

However, indirect estimates are inherently observational because individual trials are not randomised to treatment options, in contrast with the trial participants. Therefore, as with observational studies, confounding can be an issue (see below). However, it should also be noted that indirect estimates usually contribute less information if comparably sized direct evidence is available because of how variance is calculated. Detailed analyses of this issue have concluded that they are generally equally susceptible to bias except in particular circumstances.^23^

## Systematic review methods

As with PWMA, a well-defined research question is required in population, intervention, comparison, outcome, and statistical analysis (PICOS) format. In particular, included interventions should be defined *a priori,* and the authors should make explicit which interventions will be grouped and treated as identical, which we call a treatment node. Placebo trials should be included as they usually provide the largest number of studies and are a good source of indirect evidence. Indeed, exclusion of these studies can have large effects on NMA effect estimates.^24^ Also, older interventions should be considered even if they are used less frequently in clinical practice as they can provide valuable indirect evidence.^25^

Careful consideration must be taken when grouping interventions, which needs a clinical rationale. The more diverse the interventions included in a node, the greater the risk of clinical heterogeneity. However, this is offset by an increase in precision in estimates and network connectedness. The type of study for inclusion should also be considered. RCTs remain the gold standard for assessing interventions. If comparative observational studies are to be considered, specific methods have been proposed for incorporation of non-randomised evidence.^26^ Recent evidence shows that comparative observational evidence can produce similar estimates to RCTs,^27^ although this practice remains controversial.

The systematic review portion of the NMA should be conducted thoroughly by searching multiple sources and involving a clinical librarian to improve retrieval of relevant studies.^28^ This was performed in only 19% of NMAs in anaesthesia.^9^ Concerns have been raised regarding exclusion of non-English language articles causing English language bias. However, recent research concluded that this makes little difference to review effect estimates.^29^ Nevertheless, the finding that the statistical significance of results changed 9% of the time should be considered to represent an impactful influence, and therefore non-English language studies should be included.^29^

An extension of the Preferred Reporting Items for Systematic Reviews and Meta-Analysis (PRISMA) checklist called PRISMA-NMA^30^ should be used to improve the quality of reporting. However, a previous study that included more than 1000 NMAs found little effect of its introduction on the quality of reporting in NMAs over and above previous yearly increases.^31^ Poor reporting of the checklist is more common for industry-funded NMAs.^32^ Therefore, journals and peer reviewers must ensure this has been adequately completed to improve reporting, particularly as only 42% of NMAs in anaesthesia used the PRISMA-NMA checklist.^9^

As with traditional PWMA, registration of a protocol with statistical analysis, either for publication or on a database such as PROSPERO, is essential, and this is associated with higher review quality.^33^ However, <50% of NMAs in anaesthesia have a pre-registered NMA protocol.^9^ Because of the complex nature of the analysis, involvement of an experienced statistician should be implemented, although this was found in only 19% of NMAs in anaesthesia.^9^ Statistician involvement at the protocol stage will enable development of a robust statistical analysis plan.

## Quality of network meta-analyses

As with traditional PWMA, tools are available for authors to ensure that reported estimates can be presented with levels of certainty and confidence based on deficiencies in domains, as discussed in this article. An extension of Grading of Recommendations Assessment, Development and Evaluation (GRADE) is available, which incorporates issues specific to NMA.^34^ An alternative is Confidence in Network Meta-Analysis (CINeMA); this uses online software that allows data to be uploaded and automates many of the assessments required.^35^ GRADE and CINeMA can produce different ratings around a third of the time, with CINeMA being more conservative.^36^ A third option is a type of threshold analysis.^37^

Historically, reporting in NMA has been inadequate.^38^ Although the quality of NMAs has increased over the years, significant limitations still exist.^39^ A review of 438 NMAs found only a quarter were of high quality, and a fifth were of poor quality.^40^ A review of 62 anaesthetic NMAs^9^ found deficiencies in the areas of study searches, team composition, reporting of methodology, and reporting of many types of statistical analysis. Overall, 21 out of 29 reporting domains had low or very low compliance. Using the AMSTAR-2 tool, 84% of anaesthesia NMAs were rated as critically low, with quality scores increasing with journal impact factor and use of PRISMA-NMA.^41^

## Transitivity and incoherence (inconsistency)

Transitivity is the major additional assumption in NMA. It assumes that effect modifiers are equally distributed between different treatment comparisons. Alternatively, it can be defined as any eligible participant should be jointly randomisable to any of the included interventions. If this assumption is violated, avoidance of NMA or use of strategies discussed later in this section should be considered.^22^

**Fig 2:**
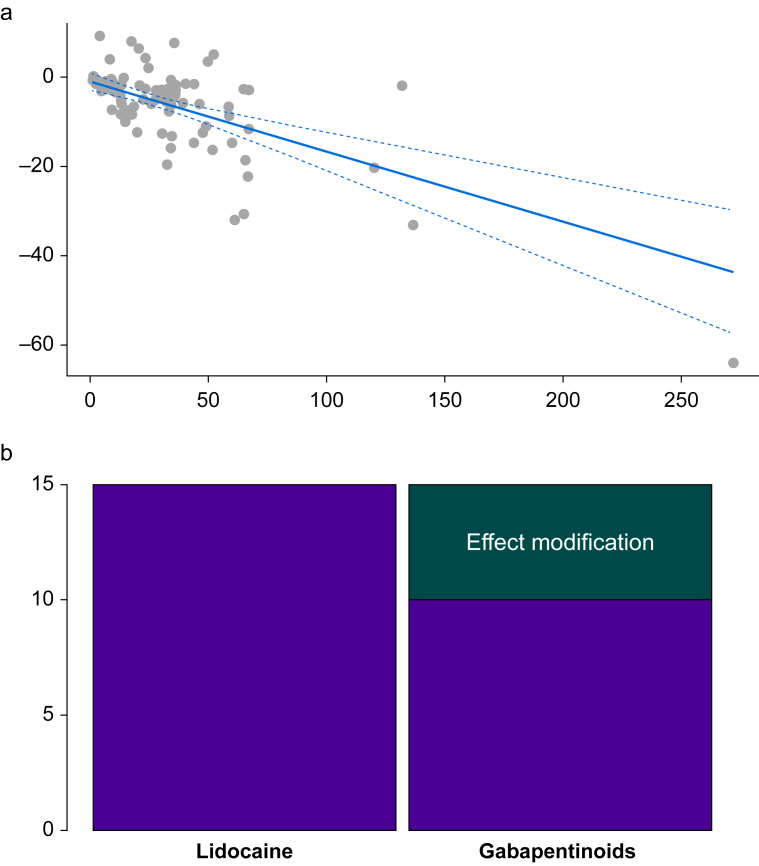
(a) Network meta-regression of α_2_ adrenergic agonists for the outcome of 24-h morphine consumption. The *x*-axis is baseline risk (control group morphine consumption), and the *y*-axis is the mean reduction in 24-h morphine consumption. As baseline risk increases, efficacy also increases. Grey dots are the effects for each study, the solid blue line is the regression line estimate, and the dashed blue line is the 95% confidence interval (CI). Clinical significance changes with the value of baseline risk (*x*), so a single conclusion cannot be drawn on clinical significance. Baseline risk is a potential effect modifier, and unequal distribution between comparisons could violate transitivity, as shown. (b) Stacked bar chart showing the absolute morphine consumption after gabapentinoids (GAB) and lidocaine (LID) relative to placebo. The purple regions highlight the true difference in morphine consumption between the GAB and LID groups (i.e. LID more effective). The green region shows the effect of a difference in the distribution of effect modifiers (baseline risk or risk of bias) between comparisons of active interventions with placebo. Despite the ‘known’ underlying benefit of LID (purple region), a difference in effect modifiers (green region) makes them falsely appear to have the same efficacy.

To illustrate this assumption using the previous example (where we assumed effect modifiers were balanced), assume that new trials of gabapentin *vs* placebo now include participants at high risk of postoperative pain (females with pre-existing pain) and all the lidocaine *vs* placebo trials include participants at low risk of pain (males undergoing laparoscopy). Because previous data have illustrated that reductions in morphine consumption are determined by the underlying risk of pain (Fig. 2a),^42^, ^43^, ^44^ it would not be valid to compare these agents indirectly under these circumstances.^45^ This is because efficacy is now determined both by the difference in efficacy between the two drugs and the difference in the distribution of this effect modifier (Fig. 2b). To demonstrate this mathematically, we assume the difference in this effect modifier causes a 5-mg reduction in morphine consumption benefitting gabapentin, which we input into equation (2):

(5) MDINDlidocainevsgabapentin=MDDIRplacebovsgabapentin–MDDIRplacebovslidocaine+Δeffectmodifierlidocainevsgabapentin=10mg–15mg+5mg=0mg

This results in no difference in efficacy in our hypothetical example where we know (unlike real life) that lidocaine results in a 5-mg reduction compared with gabapentin (2). This is termed confounding in observational evidence but also extends to NMA. A previous study has identified that re-analysing NMAs using covariate adjustment (i.e. underlying risk of pain in this context) can alter the results.^46^ Therefore, adjustment for covariates remains a practice that can mitigate inconsistency, although it can have low power if the number of included trials is low.^47^ Despite this low power, covariate adjustment should still be used because of the issue above.

Many methods have been proposed to test the transitivity assumption, each with advantages and disadvantages.^48^ However, these methods rely on knowledge of potential effect modifiers when testing the assumption and cannot account for unknown effect modifiers (‘unknown unknowns’). Before analysis, graphical displays can be used to illustrate the distribution of known effect modifiers between different treatment comparisons, similar to the contribution matrices used in CINeMA.^49^ Potential effect modifiers can also be identified using a network meta-regression using trial-level covariates. If it is found that the transitivity assumption has been violated, options include not performing an NMA or running a network meta-regression with the specific effect modifier and presenting estimates from a fixed value of this covariate.^50^ In our example, baseline risk of pain can be estimated from control group values to prevent variations violating transitivity.^12^

Although related, incoherence (inconsistency) describes statistically significant differences between direct and indirect treatment estimates. Evaluation of this requires closed loops of evidence (Fig. 1b). Using our previous example, if two RCTs were conducted comparing gabapentin and lidocaine directly and these showed a 5-mg reduction with lidocaine compared with gabapentin, then:

(6) MDINDlidocainevsgabapentin=MDDIRlidocainevsgabapentin=–5mg

Here, our indirect and direct estimates match, and consistency can be reasonably assumed. Alternatively, if the same two RCTs showed a 10-mg reduction with gabapentin, then:

(7) MDINDlidocainevsgabapentin=–5mg≠MDDIRlidocainevsgabapentin=10mg

This suggests incoherence. In practice, estimates are not from infinitely large trials and are unlikely to match exactly, so inferential statistics are performed, and consistency is assumed if a certain threshold of statistical significance is observed. However, lack of statistical incoherence does not necessarily indicate satisfaction with the transitivity assumption because of the low power of these tests.

In general, tests for incoherence can be classified as local or global. Local tests assess local loops of evidence, whereas global tests assess the network as a whole. Examples of local tests include measurements of the absolute differences between direct and indirect estimates.^51^ Direct and indirect estimates should be presented together in a forest plot to allow visual inspection for incoherence as statistical tests have low power.^52^ However, the power of these tests improves with increasing sample sizes and lower heterogeneity in the included studies.^53^ Another visual example includes net heat plots, although their reliability has been questioned.^54^ An example of a global test for incoherence is the design-by-treatment interaction.^55^ Review authors should use both approaches to inform concerns over incoherence.^19^

Because it is such an essential assumption of NMA, it is useful to estimate the incidence of incoherence in the NMA literature. A study from 2013 found an incidence of local incoherence of 2–9%.^56^ More recently, a study re-analysed 201 NMAs and found global inconsistency in up to 20% of reviews.^57^ Another older analysis of 112 trial networks reported an incidence of inconsistency of 14%.^58^ It is also useful to evaluate how often NMA authors are assessing for transitivity and incoherence. A recent analysis of 721 NMAs found that reviews were more than three times as likely to assess transitivity statistically rather than conceptually.^59^ Even within Cochrane reviews, inconsistency is only assessed around a third of the time.^60^ Another analysis of 77 NMAs found that almost half did not consider effect modification in their analyses.^61^ Similarly, around half of 438 NMAs did not assess or account for inconsistency between direct and indirect treatment effects.^40^ More specific to NMAs in anaesthesia, a recent analysis showed that transitivity was only tested in 65% of NMAs.^9^

## Risk of bias

Although we have discussed risk of bias (ROB) in underlying RCTs for PWMAs,^6^ a further consideration in NMA is the contribution that trial data make to each treatment comparison. For example, using our previous example, if all trials comparing gabapentin *vs* control are unblinded and all trials comparing lidocaine with placebo have a low ROB, this might exaggerate the effect of gabapentin, which could threaten the transitivity assumption in the same way as baseline risk. CINeMA includes online software that allows the user to upload their data to yield a contribution matrix that can identify ROB in different treatment comparisons.^35^ Because of the effects that ROB can have on treatment effects, we recommend that NMAs include only analyses with low-ROB trials, even for a sensitivity analysis.

## Heterogeneity

As with PWMA, the clinical heterogeneity of included trials should be assessed, and NMA should only be conducted if trials are sufficiently similar. It is unlikely that many RCTs from different countries and centres will have similar patient populations, which can introduce a large amount of clinical heterogeneity. This needs to be carefully considered, and if NMA is performed, random-effects models are most likely to satisfy the assumptions of included studies in a typical NMA.^6^ Clinical heterogeneity requires careful decisions on whether treatments are sufficiently similar to be included in the same group (node); this relates to, for example, different drugs with the same mechanism of action or different doses of the same drug (which can be explored using a model-based NMA^62^). This issue highlights the importance of having clinical experts as part of the review team. If combination treatments (gabapentin + lidocaine) are included, component NMA can allow estimation of the effects of combinations of interventions, even if these were not used in the original trials.^63^

Probably the most intuitive method to present statistical heterogeneity is 95% prediction intervals (PIs), which describe a range within which future treatment effects should be expected and can be used in NMA.^64^ Use of 95% PIs provides precision despite sampling error and statistical heterogeneity; they are, therefore, usually wider than the corresponding intervals based on sampling error only (confidence intervals [CI] or credible intervals [CrIs]). Usefully, these are calculated and applied automatically as part of CINeMA online software when assessing the certainty of evidence.^35^ Incoherence and heterogeneity are similar concepts; they both describe more variability than expected by chance between effect sizes. The difference is that heterogeneity describes this within the same comparison, and incoherence describes this between comparisons.

## Publication bias

Publication bias refers to the preferential publication of positive studies, which has the potential to bias the results of an NMA. Meta-epidemiological studies have identified the highly prevalent nature of publication bias within the anaesthesia literature, with an incidence as high as 43–80%.^65^ A study of the proportion of anaesthesia conference abstracts proceeding to full publication found that abstracts with positive findings were significantly more likely to proceed to publication (*P*<0.001).^66^ Mandatory trial registration on clinical trial databases can help improve the number of unpublished anaesthesia abstracts that proceed to publication^67^ and potentially reduce publication bias.^68^ However, 92% of registered RCTs in anaesthesia had an outcome discrepancy between protocols and publications (selective outcome reporting).^69^

The most important factor to reduce the risk of publication bias is a thorough search of unpublished sources.^6^ However, only half of NMAs in anaesthesia searched clinical trial databases, and only a fifth searched conference abstracts.^9^ This is also a major issue in PWMA in anaesthesia.^70^ A study from 2017 in the general literature found that only 36% of 438 NMAs assessed for possible publication bias, and only 40% of NMAs in anaesthesia assessed for publication bias via the treatment network.^9^

**Fig 3:**
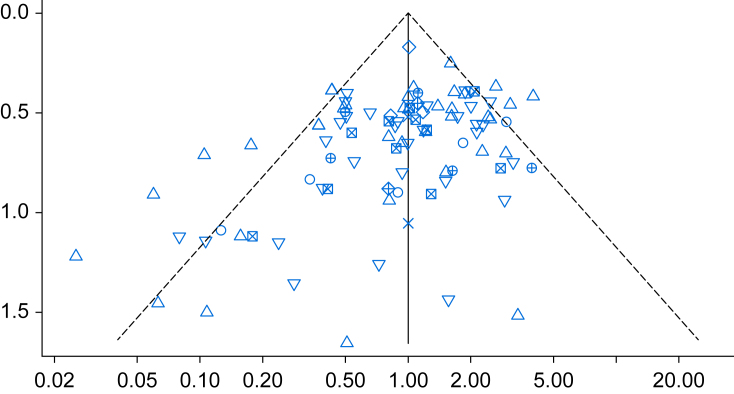
Comparison adjusted funnel plot for nonopioid analgesics in reducing the incidence of chronic postsurgical pain.^12^ The *y*-axis represents the standard error on a reverse scale, and the *x*-axis represents the effect in each study, a direct summary effect for that comparison. The specific comparison here is active *vs* control interventions, with a different symbol for each comparison (blue points). The funnel plot appears asymmetric, which is corroborated by the test for funnel plot asymmetry (*P*<0.001).

Methods for assessing possible publication bias in NMAs include selection models,^71^ comparison-adjusted funnel plots, and the approach proposed by Risk Of Bias due to Missing Evidence in Network meta-analysis (ROB-MEN).^72^ Visual inspection of funnel plots can be inaccurate and should therefore be supplemented with quantitative tests.^73^ Comparison-adjusted funnel plots (Fig. 3) are a modification of traditional funnel plots used in PWMA. However, they require a hypothesis on how publication bias manifests, such as novel *vs* old treatments. Therefore, the comparisons to be included in the plot need to be specified.

## Imprecision and random error

Power calculations are available for NMAs.^74^ Concerns over power (type II errors) and multiplicity (type I errors) occur with NMAs. This is not only because of the potential for a new analysis with the publication of a subsequent RCT, but also because of the multiple comparisons and therefore statistical tests being performed within a single NMA. A previous simulation study found that when simulating 1000 NMAs under the assumption that all treatments were equally effective, >70% of the time at least one treatment comparison demonstrated superiority.^75^

Review authors should carefully interpret effect estimates and the associated measure of imprecision. Null hypothesis statistical testing with *P*-value thresholds unfortunately leads to all-or-nothing statements on the likely effect of an intervention. Although CIs have been proposed as a preferred method,^76^ they are often used similarly to *P*-values. As an example, we use two identical effect estimates with different magnitudes of precision to highlight this important issue. For an outcome such as postoperative nausea and vomiting (PONV) with an identical intervention, one NMA has a relative estimate of 0.83 (95% CI 0.15–4.64), and another has an estimate of 0.83 (95% CI 0.79–0.88). Despite an identical estimate, the second estimate is more precise, which can be regarded as a reduction in PONV. The first estimate does not necessarily indicate ‘no difference’, rather that the estimate is too imprecise to draw any conclusions. A recent, less extreme example relates to estimates of the impact of benzodiazepines on delirium.^77^ Such assessments are usefully incorporated into CINeMA confidence estimates.

## Presentation of network geometry

The geometry of an NMA should be presented with a network plot and, ideally, supplemented with a table of network characteristics not conveyed in the plot. This can give information on the number of studies and participants, and a visual representation of the amount of direct and indirect comparisons included. A well-connected network is ideal as it provides both direct and indirect evidence (Fig. 1b), which helps in the assessment of inconsistency and can also help dilute biased estimates within a network.^78^ In addition to demonstrating network geometry,^79^ network plots can be modified to demonstrate the distribution of important effect modifiers in the evaluation of the transitivity assumption.^80^^,^^81^ A contribution matrix^82^ can also be used to identify the amount of direct evidence that contributes to each comparison.

## Presentation of effect estimates and ranking of treatments

Because an NMA can generate a number of effect estimates, presentation of these can be complicated. Because the general readership is likely to have more experience and familiarity with forest plots in PWMA, a comprehensible summary of NMA results can involve presentation of each intervention in a summary forest plot in comparison with a standard treatment node (placebo). League tables can be used to provide a matrix of effect estimates comparing all treatment combinations. In larger NMAs, these can become complicated and might be more suited to supplementary data. Variations on these can include plots for multiple outcomes,^83^ which can help balance interventions with both beneficial and harmful outcomes, forest plots displaying additional information,^84^ or plots displaying clinically significant thresholds.^85^

**Fig 4:**
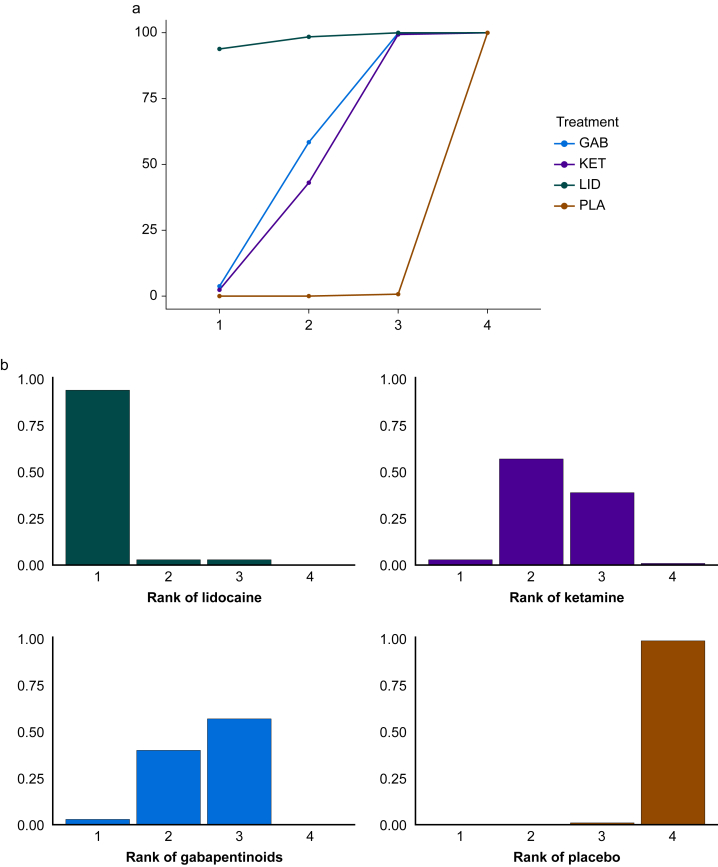
(a) Surface under the cumulative ranking curve (SUCRA) plot for the reduction in chronic postsurgical pain for lidocaine (LID), ketamine (KET), and gabapentinoids (GAB).^12^ The area under the curve normalised from 0 to 1 gives the probability that the treatment is ranked as the best (or one of the best). Lidocaine has the largest area (close to 1) and therefore is likely to be the most effective in reducing chronic postsurgical pain (CPSP). Placebo (PLA) has the lowest area under the curve and is therefore likely to be the least effective. (b) Rankogram including four nonopioids for reducing CPSP (using frequentist SUCRA simulations) showing the probability of each treatment being at a certain rank. Lidocaine is ranked first with a probability of 0.94. Ketamine is ranked at least second, with a probability of 0.63 and with a corresponding probability of 0.46 for gabapentinoids. Placebo has a probability of 1 (i.e. certain) of being ranked last.

NMA allows the ranking of different treatments. Methods to undertake this include mean/median ranks and cumulative ranking probabilities such as surface under the cumulative ranking curve (SUCRA). The SUCRA value sums all the cumulative probabilities and gives a probability for whether a treatment is among the best options (Fig. 4a). Although initially performed using Bayesian methods, it can be calculated within a frequentist framework using resampling procedures. A frequentist alternative is the P-score that gives similar results to SUCRA without resampling.^86^ These can estimate the probability that a treatment is ranked the best. Rankograms can provide a visual representation of these ranking probabilities and give a probability that a treatment is ranked at a certain position (Fig. 4b).

Although the ability to rank treatments is one of the main advantages of NMAs, such results can cause confusion and are often not presented with imprecision. For example, a previous study of 58 NMAs found that by using SUCRA, the median width of 95% CrIs was 65%, and in a third of NMAs there was a >50% probability that the best-ranked treatment was not, in fact, the best.^87^ It is therefore suggested that treatment rankings should be presented in conjunction with treatment effects and the uncertainty around both estimates.^88^^,^^89^

## Bayesian *vs* frequentist analysis

There are a range of software programmes available to assist in the performance of NMA. Packages in R can perform both Bayesian (gemtc^90^ and BUGSnet^91^) and frequentist analysis (netmeta^92^). There are also web packages that make analysis easier, without the need for coding skills (MetaInsight^93^). Frequentist statistics are commonly used in medical statistics, although criticisms persist. For example, the true definition of a 95% CI is often misunderstood by researchers; it means that if we took 100 different samples then 95% of these CIs (which would all be different) would contain the population value. Conversely, Bayesian statistics use 95% CrIs, which may be more intuitive to understand as they give probabilities that the population value is included within the interval. Although the original Bayes theorem uses prior probabilities, these are often avoided in Bayesian NMA in favour of ‘minimally informative priors’, although these are not specified in around 50% of published NMAs.^94^

Although Bayesian methods can offer more flexibility, including the range of statistical models that can be specified and estimated via Markov Chain Monte Carlo (MCMC) and related simulation methods, they require greater statistical expertise to conduct.^95^ There are specific additional statistics and plots that should be checked to ensure accuracy of the findings.^11^ Model fit should be assessed by comparing the posterior mean of the residual deviance to the number of data points. Convergence describes the point when the MCMC method reaches a stable distribution and can be assessed using trace plots and the Gelman-Rubin potential scale reduction factor. When comparing effect estimates derived from frequentist and Bayesian NMA, a simulation study found that they were generally similar in performance.^96^ The preference for Bayesian methods is reflected in there being slightly more NMAs in anaesthesia using this method (55% *vs* 40%), although detailed reporting of Bayesian model statistics occurred in only a third of reviews.^9^

## Assessing clinical significance in the presence of effect modification

Although the issue of clinical significance is not exclusive to NMA,^6^ interpretation of clinical significance in the presence of effect modification is critical. There is frequently a lack of clinical significance reported in reviews of acute pain specific to our previous examples.^97^^,^^98^ This has the potential to lead to spurious claims and might reduce the clinical use of agents that could have clinical benefit in certain circumstances. This is because whatever the covariate may be, if effect modification exists, then clinical significance varies depending on the value of the covariate. For example, in Fig 2a it can be observed that effects vary based on the covariate. Therefore, quoting a single value does not capture this nuance.^99^ Further, the use of Bayesian methods can allow probability statements on whether a treatment surpasses a minimum clinically significant threshold.

## Considerations for authors of network meta-analyses

There has been a large increase in NMA publications over recent years. It is therefore important for NMA authors to consider carefully the clinical importance of the topic and results in the review, to incorporate extensive literature searches conducted by qualified and experienced contributors, and to adhere to the highest methodological standards. In addition, the results and conclusions should be presented logically and in a format that is accessible to those reading and reviewing the article. These factors facilitate the translation of NMA results into clinical practice. In the future, artificial intelligence could help automate many of the tasks in an NMA, which is an ongoing area of research.^100^

## Conclusions

NMA offers many advantages over pairwise meta-analysis, although it is inherently more complex and has the important additional assumption of transitivity. We present evidence from published studies of violations of this assumption, coupled with inadequate conduct of NMAs in the field of anaesthesia and in the general literature. We present a methodology to help improve these weaknesses and discuss issues for the general reader to help appraise such articles in the literature.

## Authors’ contributions

Conception, data analysis: BD

Data interpretation, writing manuscript, approving final version, and agreement to be accountable for data: all authors

## Funding

NC and AS were supported by the UK National Institute for Health and Care Research (NIHR), Applied Research Collaboration East Midlands (ARC EM), and Leicester NIHR Biomedical Research Centre (BRC). The views expressed are those of the authors and not necessarily those of the NIHR or the Department of Health and Social Care.

## Declaration of interest

JH is associate editor-in-chief of the *British Journal of Anaesthesia*. The content of this article represents the experience of the authors and a presentation of the reviewed evidence. It does not represent the policy of the *British Journal of Anaesthesia*.
